# Mothers’ knowledge, attitude and home management of diarrhoea among children under five years old in Lagos, Nigeria

**DOI:** 10.4102/phcfm.v14i1.3119

**Published:** 2022-05-27

**Authors:** Faith E. Momoh, Oridota E. Olufela, Adebola A. Adejimi, Alero A. Roberts, Esther O. Oluwole, Olayinka O. Ayankogbe, Adebayo T. Onajole

**Affiliations:** 1 Department of Community Health and Primary Care, Faculty of Clinical Sciences, College of Medicine, University of Lagos, Idi-Araba, Lagos, Nigeria

**Keywords:** knowledge, home management, diarrhoea, children, mothers, Nigeria

## Abstract

**Background:**

Diarrhoea is a leading cause of death among children under five years old globally. It remains a major cause of morbidity and mortality among this age group in Nigeria. Using simple home management, mothers play important roles in the prevention and control of diarrhoea among these children.

**Aim:**

This study aimed to assess mothers’ knowledge, attitude and practice in the prevention and home management of diarrhoeal diseases among children under five years old in Lagos, Nigeria.

**Setting:**

This study was conducted within the communities of Kosofe local government area of Lagos State, Nigeria.

**Methods:**

A descriptive cross-sectional study design was conducted using a multistage sampling technique. Data were collected using a structured interviewer-administered questionnaire and analysed using EPI info version 7.2.1. Chi-square statistic was used to test the association between variable at the level of significance of 5%.

**Results:**

A total of 360 respondents participated in this study. The mean age of the respondents was 32.5 ± 5.5 years. About 59.2% of respondents had good knowledge, 59.2% of them had positive attitude, and 53.1% of them had good practice towards prevention and home management of diarrhoea. Age (*p* = 0.007), occupation (*p* = 0.008) and level of education (*p* = 0.001) were significantly associated with practice of home management of diarrhoea among children under five years old.

**Conclusion:**

Educated, employed, and married mothers were more likely to have good prevention and home management practices towards diarrhoea in their children under five years old.

## Introduction

According to the World Health Organization (WHO), diarrhoea is defined as the passage of three or more loose or liquid stools per day.^1^ It is also defined as the passage of more frequent stools than is normal for the individual.^1^ This can lead to loss of fluid, electrolytes and nutritional deficiency that can relatively progress to dehydration and death. Diarrhoea is a preventable disease, with a number of effective prevention methods.^2,3^ The prevention methods focus on some main elements, which are indicated by an acronym ‘WASH’ by the WHO meaning water, sanitation and hygiene.^1^ New aspects of this approach include rotavirus vaccination, which was recently recommended for global introduction into routine schedules for immunisation procedures, promotion of early and exclusive breastfeeding and vitamin A supplementation, promotion of handwashing with soap in terms of community-wide sanitation.^4^ It has been shown that appropriate water, hygiene, and sanitation interventions can decrease diarrhoea incidence by 26% and mortality by 65%.^5,6^

Globally, diarrhoea is a leading cause of death among children, accounting for approximately 8% of all deaths among children under five years old worldwide in 2016.^1^ This translates to over 1300 young children dying each day, or about 480 000 children a year.^2^ It kills more young children than malaria, HIV or AIDS, and measles put together.^6^ Most deaths because of diarrhoea occur among children less than two years of age living in South Asia and sub-Saharan Africa.^2^ The risk of having diarrhoea is high in developing countries.^2^ This is due to inadequate water, poor sanitation, suboptimal breastfeeding, and zinc and vitamin A deficiency.^3^ Vulnerable children living in impoverished and undeveloped areas also have higher fatality rates compared to children living in developed countries due to lack of access to quality healthcare, timely intervention, and effective treatment with oral rehydration solution (ORS) and zinc.^4^

In Nigeria, the 2013 demographic health survey revealed that 10% of children under five years old had diarrhoea in the 2-week period prior the survey.^7^ The 2018 Nigeria Demographic Health Survey (NDHS) also showed that the two weeks prevalence of diarrhoea preceding the survey among children under five years old was 13%.^8^ This makes diarrhoea a very important public health issue in Nigeria.

Diarrhoea is one of the commonest illnesses that has the greatest negative impact on the growth and development of infants and young children.^5^ Apart from diarrhoea being a major cause of morbidity and mortality, it also has a significant impact on growth due to reduction in appetite, altered feeding practices and decreased absorption of nutrients.^9^ There is a marked negative relationship between diarrhoea and physical growth and development of a child.^10^ Each day of illness due to diarrhoea produces a weight deficit of 20 g – 40 g. Infants who spend more than 20% of their time with diarrhoea have a weight deficit of approximately 370 g at follow-up after one year of age.^11^ Children with diarrhoea in the first 24 months of birth are 1.5 cm shorter than children who never had diarrhoea.^12^

Home management of diarrhoea has been recognised and advocated for by WHO, United Nations Children’s Fund (UNICEF) and Integrated Management of Childhood Illness to reduce the impact of diarrhoea, especially in children.^1,2,4,6^ Diarrhoeal diseases among children under five years old can be prevented at both primary and secondary levels by improving sanitation, water quality, early recognition of dehydration and prompt oral rehydration therapy with the use of ORS or other appropriate fluids that are available at home.^5^ Good feeding practices and better hygiene practices of children under five years old could prevent incidences of and death from diarrhoea. Other factors that can aggravate the incidence of diarrhoea in children are inappropriate feeding practices of mothers, feeding with contaminated weaning food, lack of clean water, poor handwashing or hygiene, limited sanitary disposal of waste and poor housing conditions.^5^

The aim of this study was to assess the knowledge, attitude, and practice of mothers towards prevention and home management of diarrhoea among children under five years old, in Kosofe local government area (LGA) of Lagos State, Nigeria. Specific objectives included the following:

- To determine the knowledge of mothers of children under five years old regarding diarrhoea in Kosofe LGA of Lagos State, Nigeria.
- To assess the attitude of mothers of children under five years old towards diarrhoea in Kosofe LGA of Lagos State, Nigeria.
- To assess the prevention practices and home management of diarrhoea of mothers of children under five years old in Kosofe LGA of Lagos State, Nigeria.
- To identify the factors associated with the prevention practices and home management of diarrhoea among mothers of children under five years old in Kosofe LGA of Lagos State, Nigeria.

## Methods

### Study design

This was a descriptive cross-sectional study.

### Setting

Kosofe LGA is an urban LGA located in the Northern part of Lagos State, Nigeria. It is bounded by three other LGAs namely, Ikeja, Ikorodu and Somolu LGAs. It also shares a boundary with Ogun State.^13^ It has its headquarters located at Ogudu. It is a bustling commercial city, being the terminal for most food items and fruits brought into Lagos State, from different parts of Nigeria. It serves as a place of residence for those who come in from all over the country to trade in the various markets. Kosofe also houses civil servants and government officials. The result of the 2006 national population census put the population of Kosofe LGA at 682 772, with 323 837 women,^13,14^ with a projected population of 1 321 627 in 2017.^15^ Its jurisdiction comprises 10 wards namely Agboyi, Agboyi II, Anthony/Ajao estate/Mende/Maryland, Ifako/Soluyi, Ikosi Ketu/Mile 12/Agiliti/Maidan, Isheri Olowo Ira/Shangisha/Magodo phase I & II, Ojota/ogudu, Ketu/Alapere/Agidi/Orisigun/Kosofe/Ajelogo/Akanimodo, Owode Onirin/Ajegunle/Odo Ogun, Oworonshoki and encompasses an area of about 17.85 km^2^.^13^

### Study population

The study population included mothers of children under five years old who are permanent residents of Kosofe LGA of Lagos State, Nigeria.

#### Inclusion Criteria

The study included all mothers who:

- Have lived within Kosofe LGA for at least one year.
- Gave consent to participate.

#### Exclusion Criteria

The study excluded:

- Mothers who were very ill or sick
- Mothers whose children were very ill or sick

### Sample size determination

The sample size for this study was calculated using the Cochran formula

$$\left(n={\text{Z}}^{2}\text{pq}/{\text{d}}^{2}\right),$$ [Eqn 1]

with a precision of 5% at 95% level of confidence interval. For this study, p (proportion of women with good knowledge of diarrhoeal disease) was assumed to be 62.5% based on a previous study.^5^ A minimum sample size of 360 was calculated. In order to compensate for non-response and invalid data forms, a non-response rate of 10% would be estimated. Final sample size = 400

### Sampling techniques

A multistage sampling technique was used to select respondents in the study. This was done in five different stages. Initially two wards were selected using simple random sampling from the 10 wards in Kosofe LGA. Secondly, 10 streets were selected using simple random sampling from each of the selected wards, from the list of streets obtained from the local government development authority. Thirdly, systematic sampling method was used to select 20 houses in each street after the appropriate sampling interval was calculated. Then fourthly in each house, a household was selected by simple random sampling, when more than one household met the selection criteria. However, when a selected household had no eligible respondent, another household was selected within the same house using simple random sampling. In cases where no household met the selection criteria in a house, the next house was then chosen. This continued until 20 households were selected per street. The fifth stage was the selection of respondents. Only one eligible respondent was interviewed from each household. If there were more than one eligible respondent in a household, a respondent was selected by simple random sampling. The sampling was done until the required 400 respondents were recruited to the study.

### Data collection tool

The instrument for data collection was an interviewer-administered questionnaire, adapted and modified from previous literature.^16,17,18,19^ The questionnaire contained four sections. Section A contained questions on sociodemographic status of the respondents, Section B contained questions which assessed knowledge, Section C contained questions which assessed attitude and finally Section D contained questions which assessed the practice of mothers regarding prevention and home management of diarrhoea among children under five years old.

The questionnaire was pre-tested to assess for reliability on 40 mothers of children under five years old using simple random sampling method in Surulere LGA. Surulere was chosen due to its similar demographic characteristics with the study area. The questionnaire was revised afterwards for completeness, clarity, consistency and uniformity. Content adjustments were made accordingly.

### Data scoring and grading

There were 14 questions on knowledge; some of the questions had multiple answers; 28 was the highest possible score. Each correct answer was scored 1 mark while a wrong answer was scored zero. A total score > 14 was considered as good knowledge, while a total score of ≤ 14 was considered as poor knowledge.

Nine positive and negative statements on opinion and attitude of mothers towards diarrhoea were stated. The rating scale was measured as follows: positive statement with strongly agree, agree, indifferent, disagree, and strongly disagree was scored 5, 4, 3, 2, and 1, respectively, and vice versa for negative statements. The scores ranged from 23 to 41. Each score was summed up and means of each calculated. Overall mean score was found to be 31.9 ± 3.20 standard deviation (s.d.). This was classified into two namely, positive attitude ≥ mean (32–41), and negative attitude < mean (23–31).

Eighteen questions on practice were asked. Some of the questions had multiple answers; 26 was the highest possible score. Each correct answer was scored 1 mark while a wrong answer was scored zero. A total score > 13 was considered as good practice, while a total score of ≤ 13 was considered as poor practice.

### Data analysis

Immediately after the data collection was completed, each questionnaire was thoroughly reviewed for completeness and consistency. The collected data were entered and analysed using EPI info computer software version 7.2.1 (CDC, Atlanta, GA, United States). Descriptive statistical analysis was used to compute frequency, percentages, and means of the study variables. Chi-square analysis was used to test for the association between categorical variables. The level of significance was set at 5%.

### Ethical considerations

Ethical clearance number ADM/DCST/HREC/APP/3046 was granted and obtained from the Health Research Ethics and Committee of the College of Medicine, University of Lagos, Lagos Nigeria. Informed verbal consent was also obtained directly from the respondents before the interview. This was received after the purpose of study, the envisaged benefits and harm was explained to them. Privacy was ensured during interviews. Voluntary withdrawal was also allowed for participants.

## Results

In the 20 houses surveyed, 408 mothers met all the inclusion criteria, five mothers were very ill, while 14 mothers had sick children. Twenty-nine questionnaires were incompletely filled and removed from analysis. Only 360 completely filled questionnaires were analysed.

The mean age of the respondents were 32 ± 5.5 years and 242 (67.2%) of them were within the age group of 26 and 35 years. A total of 296 (82.2%) were married, 112 (31.1%) were skilled (artisans), while 27 (7.5%) were students. Almost half 158 (43.9%) of the respondents had tertiary education and 127 (35.3%) had monthly income of N 30000.00 (Nigerian Naira) and N50000.00 ($73.00 and $121.00) at N411.00/$1.00. The predominant ethnic group was Yoruba (*n* = 200, 55.6%). Most of the respondents were Christians 212 (58.9%), and 141 (39.2%) were Muslims (Table 1).

**TABLE 1:** Sociodemographic characteristics of the respondents.

Variables	Frequency (*n* = 360)	Percentage
**Age group (years)**		
18–25	39	10.8
26–35	242	67.2
36–45	71	19.7
46–55	8	2.2
**Marital status**		
Married	296	82.2
Single	9	2.5
Widowed	46	12.8
Divorced and separated	9	2.5
**Occupation**		
Student	27	7.5
Unskilled (Petty traders, etc.)	71	19.7
Skilled (artisans)	112	31.1
Employed (office jobs)	79	21.9
Unemployed (housewives)	71	19.7
**Income (Naira)**		
N0.00–N29000.00	113	31.3
N30000.00–N50000.00	127	35.3
N51000.00–N150000.00	94	26.1
N151000.00–N350000.00	24	6.7
Greater than N350000.00	2	0.6
**Level of education**		
None	18	5.0
Primary	37	10.3
Secondary	147	40.8
Tertiary	158	43.9
**Religion**		
Muslim	141	39.2
Christian	212	58.9
Traditional	3	0.8
Others	4	1.1
**Ethnicity**		
Yoruba	200	55.7
Hausa	40	11.1
Igbo	80	22.2
Other	40	11.1

### Knowledge about diarrhoea

Table 2 shows that less than half (*n* = 145, 47.8%) knew that ORS is used to treat diarrhoea while 116 (32.2%) knew that it is not necessary to stop breastfeeding during diarrhoea episodes. More than half 252 (70.0%) knew that handwashing without soap, before preparing meals for a child can cause diarrhoea, while 231 (64.2%) knew that open dumping of faeces can cause diarrhoea. A total of 239 (66.40%) and 118 (55.2%) knew that using unclean feeding bottles for a child and contaminated food or water, respectively, can cause diarrhoea; 253 (70.3%) knew that diarrhoea is preventable and manageable at home. More than half 154 (43.0%) of the respondents attributed teething as the cause of diarrhoea while 245 (68.1%) knew that becoming weak was one of the danger signs of diarrhoea.

**TABLE 2:** Respondents’ knowledge about diarrhoea.

Variable	Frequency of correct responses (*n*)	Percentage
**The use of oral rehydration solution†**		
Rehydration	142	39.4
Diarrhoea treatment	172	47.8
No idea	45	12.5
Others	22	6.1
**It is necessary to stop breastfeeding during diarrhoea episode**	116	32.2
**Handwashing without soap before preparing food for child can cause diarrhoea**	252	70.0
**Open disposal of faeces can cause diarrhoea**	231	64.2
**The use of unclean infant feeding bottles can cause diarrhoea**	239	66.4
**Diarrhoea is preventable and manageable at home**	253	70.3
**Giving a child clean water can prevent diarrhoea**	267	74.1
**Exclusive breastfeeding for at least 6 months can prevent diarrhoea**	168	46.7
**Other methods of diarrhoea prevention (multiple responses were allowed)**		
Handwashing with soap before meal preparation	218	60.6
Early and exclusive breastfeeding	132	36.7
Vitamin A supplementation	85	23.6
Rotavirus and measles vaccination	93	25.8
Improved water supply and sanitation	81	22.5
Praying	15	4.12
Others	11	3.1
**Causes of diarrhoea†**		
Teething	155	43.1
Spiritual causes	36	10.0
Contamination	151	41.9
No idea	14	3.8
Others	4	1.1
**Danger signs of diarrhoea†**		
Becoming weak	245	68.1
Frequent passing of stool	186	51.7
Repeated vomiting	107	29.7
Fever and blood in stool	28	7.8
Marked thirst for water	32	8.9
Others	14	3.9

† , multiple responses were allowed.

### Overall knowledge towards prevention and home management of diarrhoea

Overall, 213 (59.2%) had good knowledge while 147 (40.8%) had poor knowledge about diarrhoea.

### Attitude towards Diarrhoea

A total of 307 (85.3%) agreed that diarrhoea is a serious disease that can lead to death, 247 (68.6%) agreed that it is important to handwash before preparing meals for their children, while 275 (76.40%) agreed that exclusive breastfeeding for at least the first 6 months of life is important in preventing diarrhoea. About half 189 (52.5%) wrongly agreed that giving zinc tablet during diarrhoea episodes is optional for treating diarrhoea, 222 (61.7%) agreed that ORS can be prepared at home, while 229 (63.6%) agreed that giving ORS is necessary during diarrhoea. More than half 197 (54.7%) rightly disagreed to the statement, that vaccination is not necessary for preventing diarrhoea; 206 (57.2%) rightly disagreed to the statement that, open disposal of faeces does not affect the occurrence of diarrhoea (Table 3).

**TABLE 3:** Respondents’ attitude towards diarrhoea.

Variables	SA Freq.		A Freq.		Un Freq.		D Freq.		SD Freq.	
	*n*	%	*n*	%	*n*	%	*n*	%	*n*	%
Diarrhoea is a serious disease and can lead to death	116	32.2	191	53.1	41	11.4	12	3.3	0	0.0
Infants who are bottle fed are more likely to contract diarrhoea than infants who are exclusively breastfed	74	20.6	173	48.1	71	19.7	38	10.6	3	0.8
It is important to handwash with water and soap before preparing meals for your child	109	30.3	166	46.1	59	16.4	24	6.7	2	0.7
Exclusive breastfeeding for at least the first 6 months of life is important in preventing diarrhoea	54	15.0	135	37.5	115	31.9	51	14.2	5	1.4
Giving Zinc tablet during diarrhoea episodes is optional for treating diarrhoea in children	50	13.9	155	43.1	107	29.7	47	13.1	1	0.3
Oral rehydration fluid can be prepared at home	81	22.5	141	39.2	85	23.6	49	13.6	4	1.1
Giving oral rehydration is necessary during diarrhoea	86	23.9	143	39.7	60	16.7	62	17.2	9	2.5
Vaccination is not necessary for preventing diarrhoea	33	9.2	57	15.8	73	20.3	159	44.2	38	10.6
Open disposal of faeces does not affect the occurrence of diarrhoea	23	6.4	48	13.3	83	23.1	165	45.8	41	11.4

SA, strongly agree; A, agree; Un, undecided; D, disagree; SD, Strongly disagree; Freq, frequency.

### Overall attitude towards prevention and home management of diarrhoea

Overall, 201 (55.8%) had positive attitude, while 159 (44.2%) had negative attitude towards diarrhoea.

### Preventive practices against diarrhoea in children under five

A total of 261 (72.50%) practised exclusive breastfeeding. However only 104 (28.90%) breastfed exclusively for up to 6 months. More than half 186 (51.7%) had a special place for handwashing and 274 (76.1%) washed their hands before preparing food. Most 312 (86.7%) washed their hands with soap and water, while 53 (14.7%) washed their hands with water only. Less than half 172 (47.8%) disposed their child’s faeces in the toilet, 117 (32.5%) disposed child’s faeces in the dustbin outside their houses, while 59 (16.40%) simply rinsed faeces into the gutter while washing (Table 4).

**TABLE 4:** Respondents’ diarrhoea prevention practices.

Variables	Frequency of correct responses (*n*)	Percentage
**Duration of exclusive breastfeeding**		
Less than 6 months	88	33.7
Six months	121	46.4
Others	52	19.9
**Household has a special place for handwashing**	186	51.7
**When handwashing is practised†**	7	1.9
Before food preparation	274	76.1
Before feeding children	160	44.4
After defecation	99	27.5
After attending to child who has defecated	29	8.1
**Handwashing was practised with:†**		
Water	53	14.7
Water and ash	26	7.2
Water and soap	312	86.7
Others	15	4.2
**Place of disposal of child’s faeces**		
Toilet	172	47.8
Dust bin	117	32.5
Rinsed into gutter	59	16.4
Others	10	2.8

† , multiple responses allowed.

### Home management practices for the diarrhoea

The health-seeking behaviour and feeding practices of respondents about their children, 198 (55.0%) sought help from the hospital, while 114 (31.7%), 84 (24.2%) and 39 (10.9%) sought help from the pharmacy, traditional medicine sellers and a family member, respectively (Table 5). Less than half 169 (46.9%) gave more breast milk than usual during diarrhoea, 142 (39.4%) gave the same amount of breast milk, while 35 (9.7%) gave less milk than usual. Almost half 176 (48.9%) gave more water than usual during diarrhoea, 111 (30.83%) gave same water as usual, while 60 (16.7%) gave less water than usual. Less than half 153 (42.5%) gave more food than usual during diarrhoea, while 123 (34.2%) gave less food than usual during diarrhoea episodes. Less than half 163 (45.3) had ever prepared salt and sugar solution before the study. More than half 248 (68.90%) had given ORS to their children; 142 (39.4%) also gave zinc; 159 (44.2%) also gave vitamin A; and 127 (35.3%) gave traditional or herbal medicine.

**TABLE 5:** Respondents’ health-seeking behaviour and feeding practices during diarrhoea episodes.

Variables	Frequency of correct responses (*n*)	Percentage
**Help was sought d uring diarrhoea episode from:†**		
Herbalist	84	24.2
Pharmacy	114	31.7
Hospital	198	55.0
Family member	39	10.8
Others	16	4.4
**Quantity of breast milk fed to child during diarrhoea episode**		
Less than usual	35	9.7
Same as usual	142	39.4
More than usual	169	46.9
Child does not breastfeed	7	1.9
I don’t know	7	1.9
**Quantity of water given to child during diarrhoea episode**		
Less than usual	60	16.7
Same as usual	111	30.8
More than usual	176	48.9
Nothing to drink	10	2.8
I don’t know	3	0.8
**Quantity of food given to child during diarrhoea episode**		
Less than usual	123	34.2
Same as usual	70	19.4
More than usual	153	42.5
Nothing to eat	9	2.5
I don’t know	1	0.3
Others	4	1.1
**Used oral rehydration solution (ORS) during diarrhoea episode**		
Yes	248	68.9
No	112	31.1
**Ever prepared salt and sugar solution at home**	163	45.3
**Other drugs given to child during diarrhoea episode†**		
Zinc	142	39.4
Vitamin A	159	44.2
Traditional medicine	127	35.3

† , multiple responses were allowed.

### Overall practice towards prevention and home management of diarrhoea

Overall, 191 (53.1%) had good practice, while 169 (46.9%) had poor practice towards diarrhoea prevention and home management.

### Factors Associated with Knowledge, Attitude, and Practice towards Diarrhoea Prevention and Home Management

There was statistically significant association between knowledge and all sociodemographic characteristics excluding marital status. There was a statistically significant association between attitude and level of education (χ^2^= 14.68, *p* = 0.002). There was also statistically significant association between practice and age (χ^2^ = 12.16, *p* = 0.007), occupation (χ^2^ = 13.71, *p* = 0.008), income (χ^2^ = 22.37, *p* = 0.001), level of education (χ^2^ = 37.71, *p* = 0.001), and ethnicity (χ^2^ = 8.61, *p* = 0.035) (Table 6).

**TABLE 6:** Association between sociodemographic characteristics and knowledge, attitude, and practice towards diarrhoea prevention and home management.

Variables	Frequency of respondents with good or poor knowledge						Frequency of respondents with positive or negative attitude						Frequency of respondents with good or poor practice					
	Good (*n* = 213)		Poor (*n* = 147)		*X* ^2^	*p*	Positive (*n* = 201)		Negative (*n* = 159)		*X* ^2^	*p*	Good (*n* = 191)		Poor (*n* = 169)		*X* ^2^	*p*
	*n*	%	*n*	%			*n*	%	*n*	%			*n*	%	*n*	%		
**Age (years)**																		
18–25	22	56.4	17	43.6	8.00	0.046*	11	28.2	28	71.8	2.38	0.498	12	30.8	27	69.2	12.16	0.007*
26–35	147	60.7	95	39.6			81	33.5	161	66.5			122	50.4	120	49.6		
36–45	36	50.7	35	49.3			29	40.8	42	59.2			28	39.4	43	60.6		
46–55	8	100.0	0	0.0			2	25.0	6	75.0			7	87.4	1	12.5		
**Marital Status**																		
Divorced and separated	4	44.4	5	55.6	6.19	0.103	3	33.3	6	66.7	5.30	0.151	3	33.3	6	66.7	6.84	0.077
Married	184	62.2	112	37.8			94	31.8	202	68.2			148	50.0	148	50.0		
Single	21	45.6	25	54.4			21	45.6	25	54.4			14	30.4	32	69.6		
Widowed	4	44.4	5	55.6			5	55.6	4	44.4			4	44.4	5	55.6		
**Occupation**																		
Employed	64	81.0	15	19.0	39.14	0.001*	20	25.3	59	74.7	8.53	0.074	43	54.4	36	45.6	13.71	0.008*
Skilled	76	67.89	36	32.1			34	30.4	78	69.6			64	57.1	48	42.9		
Student	13	48.2	14	51.8			12	44.4	15	55.6			10	37.0	17	63.0		
Unemployed	34	47.9	37	52.1			25	35.2	46	64.8			27	38.0	44	62.0		
Unskilled	26	36.6	45	63.4			32	45.1	39	54.9			25	35.2	46	64.8		
**Income (Naira)**																		
N0.00–N29 000.00	51	45.1	62	54.9	31.86	0.001*	46	40.7	67	59.3	5.57	0.234	36	31.9	77	68.1	22.37	0.001*
N30 000.00–N50 000.00	20	83.3	4	16.7			10	41.7	14	58.3			16	66.7	8	33.3		
N51 000.00–N150 000.00	67	52.8	60	47.2			41	32.3	86	67.7			59	46.5	68	53.5		
N151 000.00–N350 000.00	73	77.7	21	22.3			25	26.6	69	73.4			56	59.6	38	40.4		
Greater than N350 000.00	2	100.0	0	0.0			1	50.0	1	50.0			2	100.0	0	0.0		
**Level of Education**																		
None	0	0.0	18	100.0	84.15	0.001*	8	44.4	10	55.6	14.68	0.002*	3	16.7	15	83.3	37.71	0.001*
Primary	14	37.8	23	62.2			20	54.1	17	45.9			11	29.7	26	70.3		
Secondary	67	45.6	80	54.4			56	38.1	91	61.9			53	36.1	94	63.9		
Tertiary	132	83.5	26	16.5			39	24.7	119	75.3			102	64.6	56	35.4		
**Religion**																		
Christian	121	57.1	91	42.9	8.35	0.039*	66	31.1	146	68.9	3.46	0.326	107	50.5	105	49.5	0.051	0.051
Muslim	91	64.5	50	35.5			53	37.6	88	62.4			62	44.0	79	56.0		
Others	0	0.0	3	100.0			2	66.7	1	33.3			0	0.0	3	100.0		
Traditional	1	25.0	3	75.0			2	50.0	2	50.0			0	0.0	4	100.0		
**Ethnicity**																		
Hausa	20	50.0	20	50.0	14.28	0.003*	15	37.5	25	62.5	2.80	0.423	13	32.5	27	67.5	8.61	0.035*
Igbo	54	67.5	26	32.5			22	27.5	58	72.5			45	56.3	35	43.7		
Others	14	35.0	26	65.0			12	30.0	28	70.0			14	35.0	26	65.0		
Yoruba	125	62.5	75	37.5			74	37.0	126	63.0			97	48.5	103	51.5		

* , Denotes significance at *p* < 0.05.

### Multivariate analysis

Mothers who had more than six years of education, who were employed and those who were married (odds ratio [OR] = 4.52, *p* = 0.000), (OR= 2.52, *p* = 0.000), (OR = 2.12, *p* = 0.013), respectively, were more likely to have good knowledge about diarrhoea, than mothers who had six years of education or less, who were unemployed or unmarried.

Mothers who were married were 1.8 times more likely to have positive attitude towards diarrhoea, than mother who were unmarried (OR = 1.84, *p* = 0.034).

Respondents who had more than six years of education were 1.9 times more likely to have good practice towards diarrhoea than respondents who had less than six years of education.

Also, mothers who were married and those who were employed (OR = 2.03, *p* = 0.015), (OR = 1.80, *p* = 0.016), were more likely to have good practice towards diarrhoea prevention and home management than mothers who were unmarried or unemployed (Table 7).

**TABLE 7:** Multivariate analysis.

Variables	Good knowledge		*p*	Good attitude		*p*	Good practice		*p*
	Odds ratio	95% CI		Odds ratio	95% CI		Odds ratio	95% CI	
**Age**									
< or > Median reproductive years (Reference)	0.86	0.52–1.43	0.555	0.93	0.57–1.50	0.759	1.11	0.70–1.78	0.655
Median reproductive years (26–35 years)	-	-		-	-		-	-	
**Marital status**				-	-				
Unmarried (Reference)	2.12	1.17–3.85	0.013*	1.84	1.05–3.24	0.034*	2.03	1.15–3.57	0.015*
Married	-	-		-	-		-	-	
**Occupation**				-	-				
Unemployed (Reference)	2.52	1.52–4.16	0.000*	1.52	0.93–2.48	0.097	1.80	1.12–2.90	0.016*
Employed	-	-		-	-	-	-	-	
**Income (Naira)**									
Below minimum wage (Reference)	1.28	0.7433–2.2191	0.370	1.08	0.6431–0.6431	0.772	1.24	0.7447–2.0753	0.405
Minimum wage and above (≥ 30 000.00 Naira)	-	-		-	-		-	-	
**Level of education**									
Six years or less of education (Reference)	4.52	2.17–9.43	0.000*	1.90	0.98–3.69	0.057	1.90	0.97–3.71	0.060
More than six years of education	-	-		-	-		-	-	
**Religion**									
Others (Reference)	0.67	0.40–1.13	0.130	1.23	0.76–1.98	0.393	1.12	0.72–1.85	0.550
Christian	-	-		-	-	-	-	-	
**Ethnicity**	-	-		-	-				
Others (Reference)	1.75	1.08–2.84	0.023	0.72	0.45–1.15	0.172	1.45	0.92–2.29	0.107
Yoruba	-	-		-	-		-	-	

## Discussion

All respondents in this study fell within reproductive ages of 18 to 55 years. More married mothers than unmarried mothers took part in the study. This may be because married women are usually more willing to bear children than women who are not married. A higher proportion of participants were low-income earners with secondary and tertiary education. This is similar to the study done in Uyo.^19^ Participants were mostly Christians and Yoruba by tribe. This is not surprising because this is the predominant ethnic group and religion in Lagos State.

Oral rehydration solution is one of the most important medical advances of the 20th century and the cornerstone of fluid replacement.^20^ Oral rehydration solution reduces stool output and vomiting in children by 20% and 30%, respectively.^20^ It can replace water and electrolytes (sodium, chloride, potassium and bicarbonate) that are lost via liquid stools, vomit, sweat, urine and breathing.^20^ About 80% of respondents in this study knew that, ORS can be used for rehydration and treatment during diarrhoea episodes. This is less than a study conducted in Ibadan, where half of respondents rightly stated the use of ORS.^21^ But it is similar to a study conducted in Ethiopia.^22^ However, only 68% of respondents actually gave ORS to their children when they had diarrhoea. This might be due to the fact that respondents sought help in places other than hospitals, such as herbalists and family members. This is similar to a study conducted in kano where women were not using the correct method of treating diarrhoea at home by using ORS first and alone. They started off with ORS but continued to use other combination of antibiotics, and herbal medicines at various stages of the episode concomitantly with ORS.^23^

Handwashing with soap can reduce the occurrence of diarrhoeal disease, especially when carried out at critical moments, such as after using the toilet, after cleaning a child’s bottom and before handling food.^21^ Studies have revealed that handwashing with water alone is much less effective in preventing diarrhoeal disease than using soap.^24,25,26,27^ Soap is effective in breaking down grease and dirt that carry germs and disease-causing pathogens. Using soap also lengthens the amount of time spent washing hands, compared to using water alone.^24,25^ Seventy percent of the respondents knew that handwashing without soap before preparing meals for a child can cause diarrhoea. Attitude wise 46% of respondents agreed that it is important to wash hands with water and soap before meals. And as regards practice, 51% had a special place for handwashing in their houses, 76% washed their hands before food preparation, and more than 86% washed their hands with soap and water. This result is similar to a study conducted in Sudan^28^ where about 80% washed their hands before food preparation, out of whom 50% washed their hands with soap and water. But this was contrary to a study carried out in Ethiopia where only 50% of respondents washed their hands before food preparation.^29^ These discrepancies may be due to the differences in sociodemographic characteristics of participants in the different studies.

Exclusive breastfeeding is defined as no other food or drink, not even water, except breast milk (including milk from a milk bank or wet nurse) for the first six months of life, but allows the infant to receive ORS, drops and syrups (vitamins, minerals and medicines).^20^ UNICEF and WHO recommends the exclusive breastfeeding of children, because breast milk contains all the nutrients needed. Breast milk substitutes such as formula and other kinds of milk, or porridge are nutritionally inadequate and can be contaminated, thereby exposing infants to the risk of illness and mortality. Seventy-two percent of the respondents in this study breastfed exclusively, out of which only 28% did that for at least six months. This is less than a study conducted in India where of all children less than six months 56% were exclusively breastfed.^29^ It is however more than a study conducted in Benue state where the practice of exclusive breastfeeding was as low as 2%.^30^

In this study, 24% sought help from traditional healers while 55% sought help from the hospital during diarrhoea episodes. Some sought help from both hospitals and herbalists. This is more than a study carried out in Ibadan^21^ where only 4% gave native medicine. The use of traditional or herbal medicine during diarrhoea should be discouraged regardless of their availability and accessibility, because of vague dosages, and preparation of medicines under unhygienic conditions, and as evidenced by microbial contamination of many herbal preparations sold in the markets.^31^

Good feeding practices with appropriate fluids such as salt and sugar solutions, soups and weaning foods that are readily available at home can prevent dehydration and replace electrolytes that are lost during diarrhoea episodes.^5^ In this study only 42% gave more food than usual during diarrhoea episodes. Giving the same amount of foods or less during episodes may be out of ignorance and cultural beliefs in a bid to prevent more vomiting and stooling. This is similar to a study conducted in Oyo, where mothers advocated withholding food from children who had diarrhoea.^32^ Similarly, only 48% of respondents in this study gave more water than usual during diarrhoea episodes. This is not a good percentage because water makes up the greater percentage of children’s body weight. Also, children utilise more quantity of water over the course of a day, than adults as a result of their high metabolic rates. Moreover, the kidney in younger children conserve less amounts of water.^33^ This poor practice may be because only 8% of respondents knew that marked thirst for water is one of the danger signs of diarrhoea.

In this study there was statistically significant association between practice and age, occupation, income, level of education, and ethnicity. Further analysis revealed that respondents who had more than six years of education were 1.9 times more likely to have good practice towards diarrhoea than respondents who had less than six years of education. Also, mothers who were married and those who were employed were more likely to have good practice towards diarrhoea prevention and home management than mothers who were unmarried or unemployed. This is similar to a study conducted in Ethiopia^18^ where mother’s marital status and educational status were significantly associated with caregivers’ practice.

The study can be used as a baseline for other studies. There are few studies on home management of diarrhoea from the same study in the study area. Firstly, it can also be a blueprint to conduct an interventional study in the particular area. Secondly, the design was community-based descriptive cross-sectional study where probability sampling was used. One of the limitations of this study design is that the establishment of a temporal relationship between the exposure and outcome variables are impossible. Thirdly, the study area was chosen through convenience sampling as a result of time constraint.

Female education should be encouraged, because it can improve the understanding of mothers, on how to prevent and manage diarrhoea, thereby reducing the fatality of diarrhoea in children. Secondly, female empowerment should be prioritised because they are the primary care givers in a family. Women should be encouraged to get jobs and earn an income. Women who are empowered are better placed to make decisions that lead to better outcomes for their children. Finally, male involvement and support should be encouraged. Men who are involved and supportive of their wives are more likely to take better care of their children during diarrhoea episodes.

## Conclusion

A woman’s health behaviour has an immediate impact on the health of her family. Therefore, a woman’s health literacy is a crucial factor in determining her ability to adopt proper health promotion and preventive behaviours both for herself and her children.^34^

The study shows that knowledge, attitude and practice of respondents were above average. However, there were still specific poor practices such as not giving ORS during diarrhoea episodes, seeking help and advice from herbalists instead of a hospital, not washing hands with soap and water after cleaning a child who defecated, or before preparing food for the child, and giving less food and fluid than usual during diarrhoea episodes.

Mothers who had over six years of education, who were employed, or married were more likely to have good prevention and home management practices regarding diarrhoea than mothers who had six years or less of education, who were unemployed or unmarried.
